# Comparative tomographic study of the iliac screw and the S2-alar-iliac screw in children

**DOI:** 10.6061/clinics/2020/e1824

**Published:** 2020-09-03

**Authors:** Mauro Costa Morais Tavares-Júnior, Fernando Barbosa Sanchez, Jaime David Uquillas Iturralde, Renan Jose Rodrigues Fernandes, Raphael Martus Marcon, Alexandre Fogaça Cristante, Tarcisio Eloy Pessoa de Barros-Filho, Olavo Biraghi Letaif

**Affiliations:** I Departamento de Ortopedia e Traumatologia (IOT), Hospital das Clinicas HCFMUSP, Faculdade de Medicina, Universidade de Sao Paulo, Sao Paulo, SP, BR.; II Grupo de Coluna, Departamento de Ortopedia e Traumatologia, Instituto de Ortopedia e Traumatologia (IOT), Hospital das Clinicas HCFMUSP, Faculdade de Medicina, Universidade de Sao Paulo, Sao Paulo, SP, BR.

**Keywords:** S2AI, Iliac Screw, Spine Surgery, Pelvic Parameters

## Abstract

**OBJECTIVES:**

The recent advancements in spine fixation aid in the treatment of complex spinal pathologies. Both the iliac screw (IS) and the S2-alar-iliac (S2AI) screw provide adequate stability in the fixation of complex lumbosacral spine pathologies, leading to a significant increased rate of using these techniques in the daily practice of the spine surgeons. This study aims to analyze, describe, and compare the insertion and positioning parameters of the S2AI screw and IS techniques in children without spinal deformities.

**METHODS:**

An observational retrospective study was conducted at a university hospital in 2018, with 25 computed tomography (CT) images selected continuously. Mann-Whitney-Shapiro-Wilk tests were performed. The reliability of the data was assessed using the intraclass correlation. The data were stratified by age group only for Pearson’s correlation analysis.

**RESULTS:**

The mean age was 11.7 years (4.5 SD). The mean IS length was 106.63 mm (4.59 SD). The mean length of the S2AI screw was 104.13 mm (4.22 SD). The mean skin distance from the IS entry point was 28.13 mm (4.27 SD) and that for the S2AI screw was 39.96 mm (4.54 SD).

**CONCLUSIONS:**

Through CT, the S2AI screw trajectory was observed to have a greater bone thickness and skin distance than the IS. There was a linear correlation between age and screw length for both techniques. A similar relationship was observed between skin distance and age for the S2AI screw technique. In children, the S2AI screw technique presents advantages such as greater cutaneous coverage and implant thickness than the IS technique.

## INTRODUCTION

Because of the advances in spine fixation, several complex spinal pathologies can now be treated (1). Among the options available, we highlighted the Galveston technique, involving the longitudinal insertion of a bar between the two cortices of the iliac bone (2), and the iliac screw (IS) technique, where a screw is inserted in the wing of the iliac (3,4).

Compared to the former technique, the latter technique is advantageous with a greater ease of execution and increased mechanical resistance (5,6). In contrast, the disadvantages include a more prominence implant, related to the subcutaneous placement (more superficial to the skin) of the screw and to the possibility of iliac fractures (2,5,6).

Another recently developed spinopelvic fixation technique uses sacral and iliac points and is known as the S2-alar-iliac (S2AI) screw, which can even be used for minimally invasive applications (7-9). The advantages of this technique are the reduced need to dissect soft tissues, the deep positioning of the implant in relation to the skin (10), and the adequate stiffness provided by the implant position in a region of dense bone over the ischial incision (11). It has the disadvantage of the increased possibility of sacroiliac joint surface violation (7).

Both the IS and the S2AI screws provide adequate stability in the fixation of complex pathologies of the lumbosacral spine, leading to a significant increased rate of using these techniques in the daily practice of spine surgeons (3).

The aim of this study was to analyze computed tomography (CT) images of the lumbosacral area in patients without spinal deformities to determine the anatomical measurements for the insertion of screws into the pediatric pelvic spine.

## MATERIALS AND METHODS

A retrospective analysis of lumbosacral CT images from 25 continuously selected patients who underwent elective procedures at a university hospital between January and December 2018 was performed to investigate abdominopelvic pathologies (appendicitis, acute abdomen, intra-abdominal masses). The CT images and measurements were collected and analyzed using ISite enterprise software (Phillips, Amsterdam, the Netherlands). Fine-cut images with bone windows were used to obtain the measurements.

The inclusion criteria were as follows: lumbosacral CT images containing axial, sagittal, and coronal sections with the possibility of three-dimensional (3D) reconstruction; age between 3 and 18 years; and absence of bone fractures or bone tumors. The exclusion criteria were inappropriate images, incomplete records, and vertebra with congenital bone malformation or fusion defects. The study was approved by the institutional review board.

The data were stored in an Excel for Mac spreadsheet (Microsoft Corporation, Redmon, Washington). The data were entered and imported into SPSS 23 for Mac (IBM, Armonk, New York) for statistical analysis. Continuous data were described by the mean and 95% confidence interval (95% CI). Categorical data were described by the absolute frequency and respective categorical proportion. Inferential statistics were performed to compare the different sides of the body and sites in relation to the tomographic measurements. The data were tested for normality, and since no normal distribution was observed, a nonparametric paired comparison test, Mann-Whitney-Shapiro-Wilk test, was used. The intraclass correlation was used to evaluate the reliability of the data. A type I error up to 5% was accepted as a statistically significant difference. The data were stratified by age only for Pearson’s correlation analysis. Statistical analyses were performed by an independent statistician blinded to the data.

The measurements were independently obtained by four examiners. The examiners were trained to perform the measurements prior to the study, with 10 cases selected only for training and were not included in the final sample. All examiners had the same training for all measurement evaluations, and the acceptable threshold variation was 2-3 degrees for angular measurements or 2-3 mm for linear measurements. The measures were standardized as follows: age was measured in years; sex was defined as male or female; thickness, length, and width were measured in millimeters; and angle was measured in degrees.

**Figure 1:**
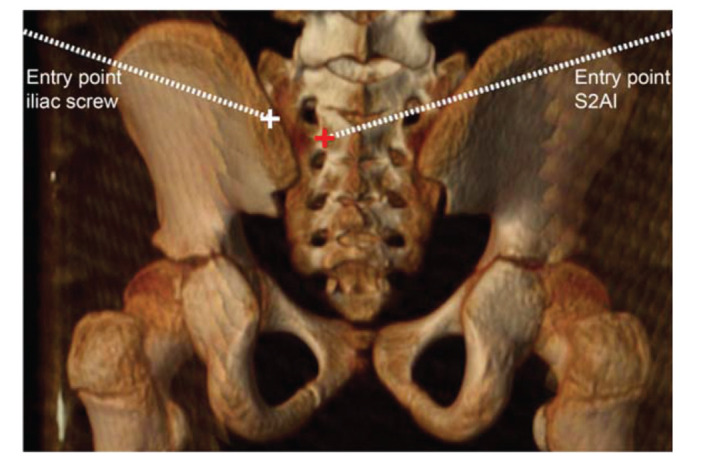
Example of the S2-alar-iliac screw (shown by the red cross) and the iliac screw (shown by the white cross) entry points.

**Figure 2:**
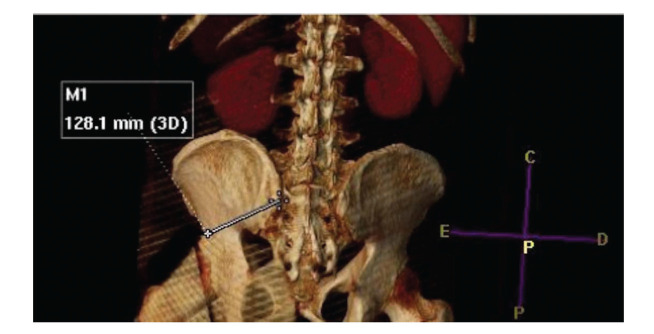
Example of iliac screw length measurement (shown by white line).

**Figure 3:**
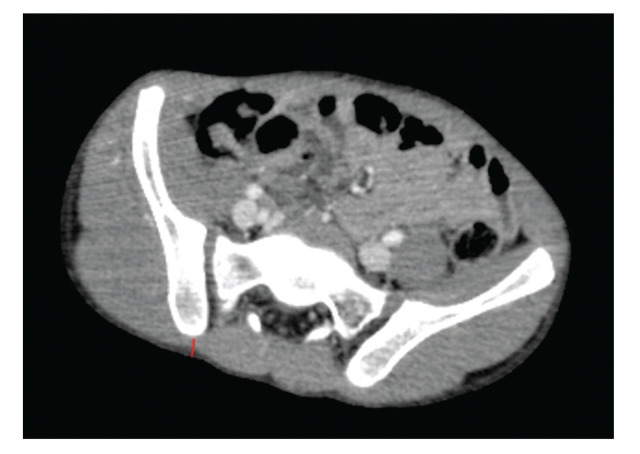
Example of distance from skin iliac screw measurement (shown by red line).

Standardization of the IS trajectory measurements (example on Figure 1):

– Length: measured from the insertion point in the posterolateral iliac crest to the anteroinferior iliac crest (example on Figure 2)
– Sagittal angle: angle of inclination of the IS in the sagittal plane of the CT
– Axial angle: angle of inclination of the IS in the axial plane of the CT
– Maximum thickness: the greatest thickness between the external cortices of the iliac crest
– Minimum thickness: the smallest thickness between the external cortices of the iliac crest in its isthmic region
– Skin distance: the perpendicular distance between the insertion point of the IS and the skin (example on Figure 3)

**Figure 4:**
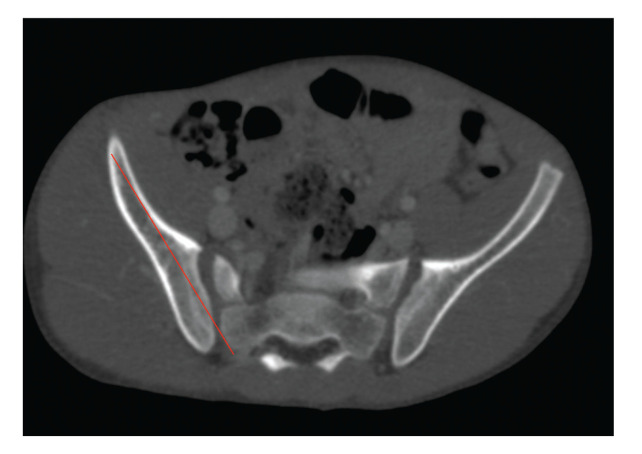
Example of S2AI screw length (shown by red line).

**Figure 5:**
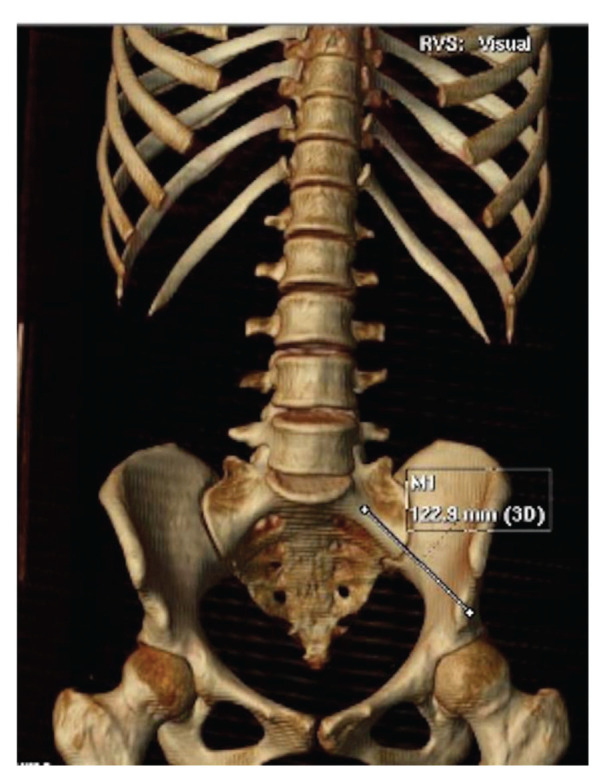
Example of S2AI screw length on CT 3D (shown by white line).

Standardization of S2AI screw trajectory measurements:

– Length: perpendicular distance between the insertion point of the S2AI screw (between the foramen of the S1 and S2 - 2 mm lateral to the paramedian sacral crest) and the anteroinferior iliac crest (example on Figures 4 and 5)
– Sagittal angle: angle of inclination of the S2AI screw in the sagittal plane of the CT
– Axial angle: angle of inclination of the S2AI screw in the axial plane of the CT
– Maximum thickness: the greatest thickness between the external cortices of the iliac crest
– Minimum thickness: the smallest thickness between the external cortices of the iliac crest in its isthmic region
– Skin distance: perpendicular distance between the insertion point of the S2AI screw and the skin

## RESULTS

We analyzed 25 CT images from 14 males and 11 females. The mean patient age was 11.7 years (4.5 SD). The mean IS length was 106.63 mm (4.59 SD). The mean length of the S2AI screw was 104.13 mm (4.22 SD). The mean maximum bone thickness of the IS was 22.62 mm (0.65 SD) and that of the S2AI screw was 23.77 (0.73 SD). The mean minimum bone thickness of the IS was 14.02 mm (0.54 SD) and that of the S2AI screw was 18.18 mm (0.64 SD).

The mean axial angle of the IS entry point was 24.06 (0.54 SD) and that of the S2AI screw was 31.96 (0.58 SD). The mean sagittal angle at the entry point of the IS was 29.86 (1.00 SD), and the S2AI was 30.50 (1.11 SD). The mean skin distance from the IS entry point was 28.13 mm (4.27 SD) and that of the S2AI screw was 39.96 mm (4.54 SD).

Virtually all of the analyzed variables were significant different between the IS and S2AI screw, except the sagittal angle. The analysis is shown in Table 1.

The intraclass reliability analysis demonstrated an excellent reliability in all measurements among the examiners. The analysis is shown in Table 2.

When the data were stratified by age, the screw length of both the IS and S2AI screw showed a linear increase with age. The skin distance of the S2AI screw also increased with age. The skin distance of the IS presented a tendency to increase with age (*p*>0.05 but <0.10). However, the other data analyzed did not correlate with age (Table 3).

## DISCUSSION

In this study, the length of the screw trajectory, skin distance, maximum and minimum thickness, and axial angle were different between the IS and S2AI screw in children.

The IS technique and S2AI can be used both in adults or children, but the pathologies where both techniques are used vary depending on the population and their most prevalent problems. The skeletal anatomy of children may have several variations depending on the age group studied, therefore, studying the differences between the techniques in different age groups is necessary compared to adults.

Considering the trajectory of the screw, the IS was about 2.5 mm larger than the S2AI screw. Without considering the screw trajectory, both the maximum and minimum thickness of the IS (1.15 mm and 4.16 mm, respectively) were higher than those of the S2AI screw. This can be justified by the difference in pelvic inclination and the volume between the upper and lower parts of the pelvic region or due to comparison in the differences between the expected trajectories without considering difficulties of execution between the techniques, generating values different than those seen in practice.

Several authors have described similar results in the sagittal and axial angles (12). The screw trajectory angles present considerable variations, reaching up to 15 degrees in the sagittal plane (12,13).

Another important consideration regarding the surgical and postoperative parameters is the distance of the implant from the skin. The IS had a mean distance of 28.13 mm, and the S2AI screw 39.97 mm. Thus, some of the fixation problems in the lumbosacral region are prominence of the synthesis material, minimal skin coverage, pelvic morphological change, possibility of failure of the implants, bone fracture, among others (2,4,5,6). Therefore, this difference results in a deeper implantation into the soft tissues and a lower risk of exposing the synthetic material. This finding has enhanced importance in the pediatric population, since exposure often occurs in patients with low body mass index, cognitive deficiencies, and cutaneous insensitivity, as well as patients on wheelchairs or those who are bedridden.

Although the data of this study and that from the literature are in excellent agreement, there are some numerical differences that can be explained by the different entry points for the two techniques, since even a few millimeters can significantly alter the final values (3). However, these differences are not necessarily clinically relevant despite being significant.

In this study, possible biases may arise from the retrospective nature of the study and from using multiple examiners. However, the biases were minimized through prior training and the standardization of measurements, which can be observed from the excellent overall reliability of the measurements (Table 2). Differences of 2-3 mm or 2-3 degrees are not clinically significant.

There were no significant differences between sex and laterality. Thus, the mean of the right and left sides was used to obtain the statistical power of the sample.

Although it was not the focus of the study, the finding of the S2AI screw affecting pelvic balance should be emphasized, as this modifies the value of the S2AI screw (14,15), and this finding may be clinically relevant, especially in a pediatric population with immature skeletons. Pelvic incidence is considered a constant morphological parameter after skeletal maturity. However, some recent studies have questioned this fact (16,17). These aspects deserve further investigation.

## CONCLUSIONS

Through CT, the trajectory of the S2AI screw was observed to have a greater thickness and skin distance than the IS. A linear correlation between age and screw length was observed for both techniques. A similar relationship was found between age and skin distance for the S2AI screw technique. In the pediatric population, the S2AI screw technique presents advantages of greater cutaneous coverage and implant thickness than the IS technique.

## AUTHOR CONTRIBUTIONS

Tavares-Júnior MCM, Sanchez FB, Iturralde JDU, Fernandes RJR reviewed the literature, collected, and analyzed the data and wrote the manuscript. Marcon RM, Cristante AF and Barros-Filho TEP analyzed the data, final reviewed the literature, and were responsible for the project and manuscript. Leitaf OB designed the study, analyzed the data, final reviewed the literature, and was responsible for the project and manuscript.

## Figures and Tables

**Table 1:** Mann-Whitney and Shapiro-Wilk tests.

Data	Iliac	S2AI	Mean difference iliac x S2AI	Standard deviation	95% confidence interval (CI) - superior limit	95% confidence interval (CI) - inferior limit	*p-*value
Screw length	106.63	104.13	2.50	4.18	4.23	0.77	<0.01
Maximum bone thickness	22.62	23.77	-1.15	1.40	-0.57	-1.72	<0.01
Minimum bone thickness	14.02	18.18	-4.16	1.41	-3.58	-4.75	<0.01
Axial screw angle	24.06	31.96	-7.90	1.33	-7.35	-8.45	<0.01
Sagittal screw angle	29.86	30.50	-0.63	3.22	0.70	-1.96	0.341
Distance from skin	28.13	39.96	-11.84	5.26	-9.66	-14.01	<0.01

**Table 2:** Comparison of the intraclass correlation coefficients.

Data	Intraclass correlation*	95% confidence interval (CI) - superior limit	95% confidence interval (CI) - inferior limit
Screw length			
Iliac	0.984	0.992	0.971
S2AI	0.951	0.976	0.913
Maximum bone thickness			
Iliac	0.840	0.917	0.732
S2AI	0.848	0.922	0.745
Minimum bone thickness			
Iliac	0.771	0.878	0.631
S2AI	0.774	0.879	0.635
Axial screw angle			
Iliac	0.774	0.862	0.594
S2AI	0.767	0.876	0.626
Sagittal screw angle			
Iliac	0.798	0.893	0.670
S2AI	0.756	0.869	0.611
Distance from skin			
Iliac	0.972	0.986	0.950
S2AI	0.954	0.977	0.918

* Interpretation: less than 0.400: poor; 0.400-0.599: fair; 0.600-0.749: good; and 0.750-1.00: excellent.

**Table 3:** Comparison of the Pearson’s correlation Coefficients.

Data	Pearson’s correlation coefficient	*p*-value
Screw length		
Iliac	0.915	<0.01
S2AI	0.904	<0.01
Maximum bone thickness		
Iliac	0.545	0.005
S2AI	0.593	<0.01
Minimum bone thickness		
Iliac	0.694	<0.01
S2AI	0.796	<0.01
Axial screw angle		
Iliac	-0.330	0.107
S2AI	-0.243	0.242
Sagittal screw angle		
Iliac	0.059	0.778
S2AI	0.008	0.971
Distance from skin		
Iliac	0.345	0.091
S2AI	0.466	0.019
